# Tumors Are Evolutionary Island-Like Ecosystems

**DOI:** 10.1093/gbe/evab276

**Published:** 2021-12-11

**Authors:** Antonia Chroni, Sudhir Kumar

**Affiliations:** 1 Institute for Genomics and Evolutionary Medicine, Temple University, USA; 2 Department of Biology, Temple University, USA; 3 Center for Excellence in Genomic Medicine Research, King Abdulaziz University, Jeddah, Saudi Arabia

**Keywords:** cancer evolution, metastasis modeling, tumor biogeography, tumor island

## Abstract

Integration of ecological and evolutionary features has begun to understand the interplay of tumor heterogeneity, microenvironment, and metastatic potential. Developing a theoretical framework is intrinsic to deciphering tumors’ tremendous spatial and longitudinal genetic variation patterns in patients. Here, we propose that tumors can be considered evolutionary island-like ecosystems, that is, isolated systems that undergo evolutionary and spatiotemporal dynamic processes that shape tumor microenvironments and drive the migration of cancer cells. We examine attributes of insular systems and causes of insularity, such as physical distance and connectivity. These properties modulate migration rates of cancer cells through processes causing spatial and temporal isolation of the organs and tissues functioning as a supply of cancer cells for new colonizations. We discuss hypotheses, predictions, and limitations of tumors as islands analogy. We present emerging evidence of tumor insularity in different cancer types and discuss their relevance to the islands model. We suggest that the engagement of tumor insularity into conceptual and mathematical models holds promise to illuminate cancer evolution, tumor heterogeneity, and metastatic potential of cells.

Significance

The integration of ecological and evolutionary aspects holds promise for understanding cancer evolution and metastatic potential. Here, we suggest that tumors are evolutionary island-like ecosystems, that is, isolated evolutionary ecosystems with their heterogeneity shaped by features such as physical distance and connectivity between anatomical sites. We suggest that these features dictate the evolutionary and spatiotemporal dynamic processes in tumors and drive the migration of cancer cells. The analogy of tumors as insular evolutionary systems is a conceptual framework that can leverage extensive genetic and epigenetic aberrations observed in the genomes of cancer cells. The tumor island framework will enable us to explore the relationship between tumor heterogeneity and microenvironment and illuminate the metastatic potential of cells.

  “Islands are reminders of arrivals and departures.”

           Ehrlich (1992)

## Background

Cancer cells can move, spread, and form metastases at proximate and distant locales in the body (Williams et al. 2019). The formation of tumors is nothing like walking along a straight line but rather dwelling at the center of the Minotaur’s labyrinth. A cancer cell leaving its initial tumor of growth needs to navigate the complex maze of blood and lymphatic stream to arrive at a distant anatomical site where it faces an uncertain fate to invade, survive, and colonize the new environment in the process of forming a metastasis. The ability of cancer cells to move and form metastases depends on a host of evolutionary and spatiotemporal processes that affect tumor heterogeneity and the microenvironment. The size and grade of a tumor, its spatial and temporal isolation, and tumor interconnectivity are well recognized for their contributions during disease progression, but they are poorly integrated into a framework for exploring and modeling tumor dynamics and metastatic potential (Chen et al. 2011; Zhang et al. 2016; Abbosh et al. 2017; Turajlic, Xu, Litchfield, Rowan, Chambers, et al. 2018; Hayashi et al. 2020).

We suggest that tumors are evolutionary island-like ecosystems (islands hereafter) that experience insularity and evolution like true islands inhabited by biological organisms. In this framework, molecular, histological, and morphological heterogeneity within and among tumors is a response to distinct features of insularity, such as physical distance, connectivity, and species–area relationships. These features are fundamental properties that shape the diversity of islands experiencing founder effects, waves of migrations, and organismal evolution. We propose that similar features dictate tumor heterogeneity, drive migration of cancer cells, and lead to metastases. This is because there is emerging evidence that new tumors are seeded by one (Turajlic, Xu, Litchfield, Rowan, Chambers, et al. 2018) or more clones (Macintyre et al. 2017; Noorani et al. 2020) from primary and other pre-existing tumors, and there is reseeding of clones (Norton and Massagué 2006; Kim et al. 2009; Savas et al. 2016; Yates et al. 2017) as well as exchange between tumors (primary-to-metastasis and metastasis-to-metastasis spread) (Macintyre et al. 2017). This is similar to migrations between islands in organismal biogeography, although the number of clones migrating between islands at any given time may be only a few (Birkbak and McGranahan 2020).

The analogy of tumors as evolutionary insular systems is a unique, conceptual approach that needs to be explored and integrated into mathematical and computational models being developed to study tumor evolution. Our proposed conceptualization follows the recent application of organismal biogeographic approaches into the investigation of cancer cells movements in a patient (Alves et al. 2019; Chroni et al. 2019, 2021). We provide initial evidence that supports the proposed analogy of tumors as islands, present fundamental hypotheses and predictions, and discuss the powers and pitfalls of this analogy. In addition, we suggest adopting the “immigration” thinking from organismal biogeography together with the clonal evolution and the tumor microenvironment formation as an explanatory variable of tumor heterogeneity.

## Tumors Are Products of Evolution

Theodosius Dobzhansky once said that “nothing in biology makes sense except in the light of evolution” (Dobzhansky 1973), which has been the inspiration for declaring “nothing in *cancer* makes sense except in the light of evolution” (Greaves 2018).

Cancer in a patient represents a continuum of evolutionary processes in which all the genetic variation traces back to one cancer cell formed through mutations of a normal cell (Nowell 1976). Cancer cells evolve continually over a person’s lifetime, causing extensive intra and intertumor genetic heterogeneity in primary and metastatic tumors (Williams et al. 2019). Unraveling cancer’s evolutionary processes hold promises for more effective biomarkers and therapeutic treatments (Gatenby and Brown 2020). There is now a growing recognition that the principles and concepts of molecular evolution and population genetics are needed for deciphering the genetic and epigenetic changes found in the genomes of cancer cells (Somarelli et al. 2020). Tumor heterogeneity can be measured by the distinct number of cell or clone types, different molecular signatures, and standard genetic diversity in tumors (Williams et al. 2019).

Genetic and genomic variation present in cancer cells is a valuable tool for investigating evolutionary processes and patterns in tumors. Indeed, many researchers have begun to employ multidisciplinary frameworks for inferring evolutionary relationships, origins, and movements of cancer cells between tumors (El-Kebir et al. 2018; Miura, Gomez, et al. 2018; Kumar et al. 2020; Somarelli et al. 2020). These trends establish the importance of studying genetic heterogeneity by integrating frameworks and models from the fields of tumor molecular evolution along with organismal ecology and biogeography. Researchers are investigating the heterogeneity and migration of tumor clones through methods designed to study the diversity and structure of species community and infer species phylogeographic and biogeographic patterns (Maley et al. 2006; Martinez et al. 2016; Alves et al. 2019; Chroni et al. 2019, 2021). These efforts suggest that tumors as islands analogy can provide insights into cancer initiation, dynamics, evolution, and metastasis.

## Tumors Are Products of Their Microenvironments

Cancer cells restructure and alter their microenvironments throughout a tumor’s evolution and even create their own ecosystem (Qian and Akçay 2018). The tumor’s ability to engineer the cell’s microenvironment extrinsically influences tumor heterogeneity and is the key component of tumor’s fitness and survival, for example (Yang et al. 2014).

Moreover, metastasis is a matter of cell dissemination and invasion and a process requiring the construction of a pre-metastatic niche that makes metastasis a product of niche construction or ecological engineering (Qian and Akçay 2018; Solary and Lapane 2020). In an ecological niche, individuals share and compete for resources, both being crucial for migration. The niche construction by metastatic processes is related to cancer cells’ ability to change their microenvironment and whether and how the modulation of the tumor’s environment will promote the propagation of cancer cells in localized, regional, or distant organs. Changes in the genomes of cancer cells over time shape the tumor’s microenvironment and niche by modifying the extracellular matrix and cellular signaling pathways and remodeling the vasculature (Sleeman 2012; Halama et al. 2017; Akhtar et al. 2019).

From an ecological perspective, the human body has been considered a landscape with heterogeneous patches that are habitats with different capacities for supplying and sustaining diversity (Maley et al. 2017). In ecology, landscapes are defined by spatially heterogeneous patches, leading to landscape mosaics (Daoust et al. 2013). The composition and configuration of these patches create biological diversity (Wiens 2008) associated with genetic variations and environmental conditions (Balkenhol et al. 2017). The knowledge of landscape architecture can result in a better understanding of the relationship of the co-inhabitants, their potential competition for limited resources, and their survival ability. Landscape architecture can also inform about turnover, that is, the rate at which clones go extinct and new clones are formed—a notion based on MacArthur and Wilson’s theory of island biogeography (first discussed in 1967)—and, ultimately, to the “pressure” to migrate (MacArthur and Wilson 2016).

In cancer research, it is though that the heterogeneity observed across the landscape of the tumor microenvironment is involved in the evolutionary and metastatic processes because it may cause the dispersal of cancer cells (Tissot et al. 2019). Considering a tumor as a niche has also led to integrative frameworks that merge ecological and evolutionary approaches for inferring origins of cancer cells and explaining reasons for therapy resistance and treatment failures (Maley et al. 2017; Thomas et al. 2020). We now need concepts, models, and computational approaches that consider migration, isolation, and connectivity between anatomical areas that are sources and recipients of metastases.

## Tumors Are Islands

**Fig. 1.**
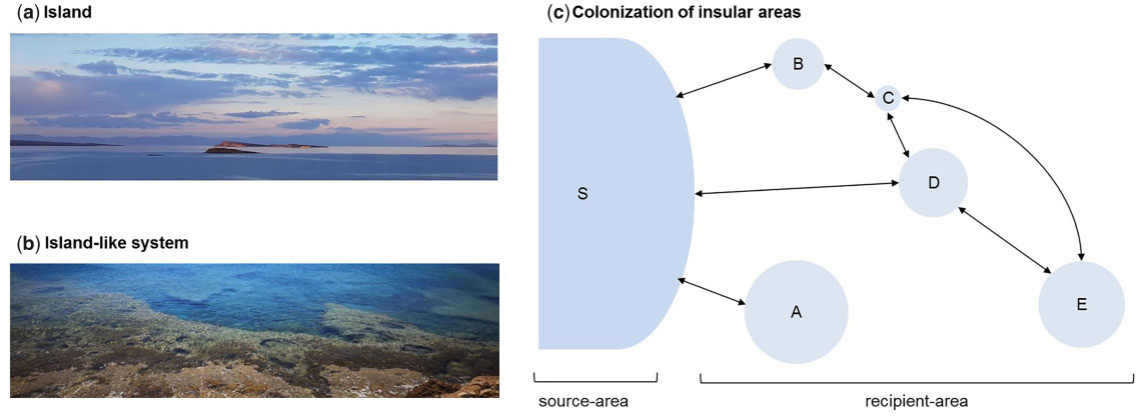
(*a*) Example of a true island (islets close to Lesbos Island, Greece). (*b*) An island-like system (reef coast in Ano Koufonisi Island, Greece). (*c*) Colonization of insular areas is the result of migrations between source and recipient areas. Size of recipient areas and distance and connectivity between the site of origin of the migration to recipient area, that is, from the source to recipient area and between recipient areas matter for successful colonization. Here, we present an example of a network between source (S) and recipient areas (A–E). In this network of insular systems, we observe insular areas of different sizes and their potential intraconnections. The size of the circle is proportional to the size of the areas. Arrows show migration can happen back and forth between areas. Photo credit: A. Chroni (2019).

On earth, islands are landmasses surrounded by water. They are disconnected and isolated to varying degrees from adjacent systems (fig. 1*a*). Isolated systems sharing these characteristics can be conceptualized as islands. They are known as “island-like” systems (ILSs) or “biological” islands, such as the reef coast shown in figure 1*b*. ILSs are characterized by spatial fragmentation and limited area, resulting in fewer available niches and lower habitat diversity. Moreover, there might be spatial and temporal isolation of areas, which function as a supply for colonization, due to low connectivity between areas (Itescu 2019). Several factors dictate diversity within an island and ILS. They include 1) the relationship between the size of the island and its connectivity to adjacent areas; 2) the species composition (which is affected by seasonality), and the evolution of new populations and species radiations besides the presence of endemic species; and 3) the degree of genetic divergence of the species (Rosindell and Phillimore 2011; Borregaard et al. 2017; Ren et al. 2017; Itescu 2019; Matthews et al. 2019).

We suggest that individual tumors experience isolation similar to islands and should be regarded as islands and ILSs. The cartoon in figure 1*c* shows an example of a network of islands that consists of a source area (S) and recipient areas (A–E) experiencing insularity. In this network of insular systems, we observe the colonization of insular regions of different sizes and their potential interconnections. Translating this to cancer, the primary and metastatic tumors can be both source and recipient areas depending on whether it is the starting or ending point of colonization of cancer cell lineages. In this case, connections between source and recipient areas are revealed through primary-to-metastasis and metastasis-to-metastasis migrations and spread. Also, tumor insularity occurs because the tumor microenvironment is a function of the size of the anatomical area and the availability of resources (Chen et al. 2011; Van Zijl et al. 2011; Wai et al. 2013). Traits shaping tumor insularity showcase the imprint of evolutionary processes and genomic variations that drive the migration of cancer cells.

Physical distance and connectivity between source and recipient areas are expected to impact dispersal and extinction rates and, ultimately, the migration fate of cancer cells (Acevedo et al. 2015; Pein and Oskarsson 2015). One may reasonably assume that organs better connected or close to the organ with the primary tumor are more likely to receive metastatic cells. For example, blood vascularization is key for cancer cells to migrate and colonize distant organs, making the consideration of connectivity between two organs as a model parameter important when studying metastatic disease (Liu et al. 2017; Pereira et al. 2018; Palazzolo et al. 2020). The size of the recipient areas will also affect the migration of cancer cells in the body because small and large areas differ in their carrying capacities and resources available in ways similar to islands (Sanmartín et al. 2008).

That being said, two theories have been proposed for understanding the metastatic propensity of cancer cells. One theory postulates that cancer cells disseminate randomly within the body following blood flow distribution (Virchow 1856) and are mechanically arrested in the first tissue they encounter (Ewing 1928). On the other hand, the “seed and soil” theory focuses on the perfect candidate organ being the recipient area as a necessity for a metastatic malformation to start (Paget 1889). Cancer cells are seeds that need a specific environment (soil) to proliferate and form a metastasis. This hypothesis has been used to explain metastatic propensities in certain types of cancer (De Groot et al. 2017).

In reality, these two theories are not mutually exclusive because both mechanical obstruction and chemical signals secreted in specific organs likely cause cells to land in a distant organ where the environmental conditions will dictate tumor growth and survival (Chu and Allan 2012). This mutual metastatic theory is supported by circulating tumor cells (CTCs) in the blood of patients with metastatic tumors. CTCs can colonize new anatomical sites contributing to cancer progression (Galizia et al. 2013; Ulz et al. 2017). Moreover, CTCs of certain cancers show a preference for tissue to form metastases, a phenomenon known as organ tropism (Gao et al. 2019). For example, prostate cancer almost exclusively metastasizes in the bones (De Groot et al. 2017), whereas ovarian cancer often metastasizes in the abdominal cavity (Kerr et al. 2015; Gao et al. 2019; Fares et al. 2020). Directionality and preference during the metastatic processes suggest selective pressures, but also presence of tumor insularity, because such tendencies modulate patterns of cancer cell spread (Kerr et al. 2015; Gao et al. 2019; Fares et al. 2020).

## Modeling Tumors and Tumor Heterogeneity as Evolutionary Islands

Integrating an ecological view into our understanding of cancer cells’ evolutionary dynamics has undoubtedly stimulated our thinking of tumors as ecosystems that undergo evolutionary and spatiotemporal dynamic processes (Maley et al. 2017; Thomas et al. 2020). Although this integration has been exciting, it is missing important features such as insularity and migration rates that are key to describing the ability of cancer cells to migrate and metastasize. We consider tumor heterogeneity to be analogous to the species diversity found on islands. We also regard physical distance and connectivity between tumors and the formation of the tumor’s enclosed microenvironment (related to limited size area and availability of resources) relevant to cancer cell (and organismal) migrations.

**Fig. 2.**
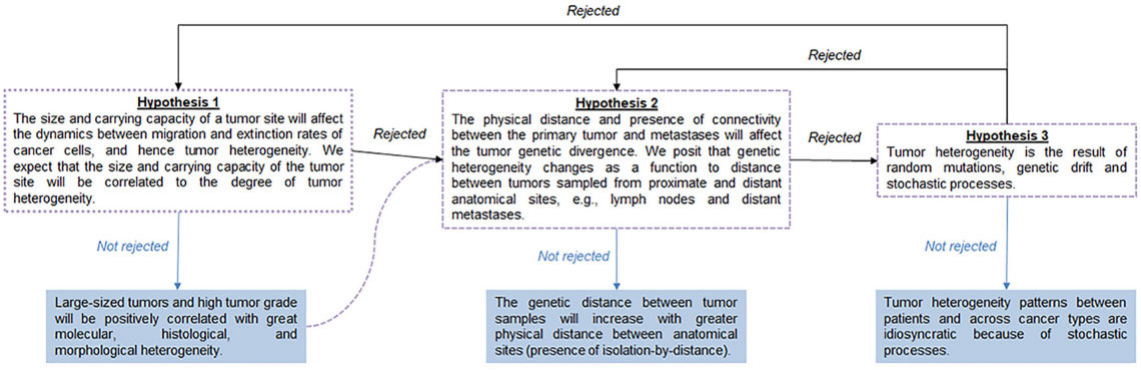
—Flowchart of the hypothesis testing of tumors as evolutionary islands. Rejection of hypotheses #1 and #2 might lead to the validation of hypothesis #3. Confirmation of hypothesis #1 does not necessarily reject hypothesis #2 because tumor heterogeneity patterns could still result from peculiar relationships between cancer cells and tumor sites and the degree of physical distance between anatomical sites. Tumor heterogeneity can be measured in numbers of cell or clone types, distinct molecular signatures, and average heterozygosity across sites, and standard genetic diversity in tumors. Ultimately, all three hypotheses may be true, depending on the cancer type and the number of tumor sites in a patient.

Therefore, we suggest that principles, concepts, and approaches of island biogeography will be useful for modeling and exploring tumor heterogeneity. We consider that the theory of island biogeography and its predictions in equilibrium can provide a framework to make a variety of predictions and formulate hypotheses about tumor heterogeneity. This is because this theoretical framework enables predictions about species diversity and populations’ fate by considering available areas and resources for colonization of species as well as distance and connectivity between locations. We can test hypotheses and shed light on the expected tumor heterogeneity because of the tumor’s evolution and isolation at spatial and temporal scales. Such a framework would account for the relationship between tumor sites’ size and carrying capacity, the degree of physical distance and connectivity between anatomical sites, and the effect of random mutations and genetic drift due to stochastic processes (fig. 2).

In considering tumors as evolutionary islands, the first hypothesis is the relationship between a tumor site’s size and carrying capacity with overall tumor heterogeneity (fig. 2). This is because the theory of island biogeography assumes a dynamic relationship between the number and diversity of species and the size of the island area. More specifically, species diversity in equilibrium is expected to correlate with the size of the island positively. A large-sized island is expected to harbor high species diversity, whereas a small island will have restricted species diversity because of limited area and resources.

In the tumor island analogy, we would predict that tumors’ genomic, histological, and phenotypic heterogeneity will be proportional to the tumor site’s size and carrying capacity. Indeed, there is evidence that large tumors are more heterogeneous in lung, pancreatic, and renal cancers (Hatt et al. 2011; Kuo et al. 2017; Li et al. 2018; Sugiyama et al. 2019; Payan et al. 2020). These studies report positive correlations between tumor size and lymph node metastasis incidence, tumor progression, overall survival, and poor prognosis.

The equilibrium model of island biogeography also predicts that species diversity will be negatively correlated with the distance between the main source area (mainland) and the recipient area (island). Islands closer to the mainland harbor higher diversity than islands at a greater distance from the mainland. This is because new species are introduced to the adjacent island constantly (continually), keeping a high dispersal rate between the mainland–island ecosystems. This prompts the second hypothesis about the possible effect of physical distance between the primary tumor and subsequent metastases and its impact on tumor genetic divergence (fig. 2). Under this hypothesis, tumor genetic divergence will be a function of the physical distance and connectivity between the anatomical sites of tumors. For example, we anticipate that there will be a higher likelihood of cancer cells to migrate to adjacent locations, which would potentially homogenize the clonal diversity present in both areas.

The second hypothesis can be tested through the presence of isolation by distance (IBD) (Wright 1943) and of physical barrier between tumors. IBD investigations help to understand if the population structure results from ongoing gene flow or other dispersal pathways (Slatkin 2017). The presence of IBD in tumor data could be addressed by investigating genetic heterogeneity changes with the distance between tumors sampled from local, regional, or distant anatomical sites spatially isolated from each other. In the presence of IBD, we expect the genetic distance between tumor samples to increase with the greater physical distance between anatomical sites. Validation of this hypothesis will indicate that physical distance between tumors has a strong effect on tumor heterogeneity.

On the other hand, a low genetic distance between distant tumor samples will show the absence of IBD and no effect of physical distance on tumor heterogeneity. We also expect low or high tumor heterogeneity to be the result of the number of clones (single or multiple) and the presence of reseedings involved in the formation of tumors, because this type of migrations would contribute to tumors net genetic diversity. Considering the type and number of seedings between tumors will be critical when interpreting inferences related to the effect of physical distance on the overall tumor heterogeneity. Findings related to the presence or absence of IBD in tumors will be exciting as they would provide insight into the relevance of tumor heterogeneity to the specific tissues and organs where tumors manifest (e.g., organ tropism) and indicate molecules or mechanisms specifically found there that might be contributing to tumor heterogeneity and driving cancer cell migrations.

In addition, we expect intertumor heterogeneity to be primarily a function of past evolutionary events because clones that seeded a metastasis, and further diversified within tumors, will always have a much larger intertumor difference than clones that have evolved within tumors. This is similar to the expectation for natural populations in which divergence and diversity are thought to be primarily a result of past spatial isolation caused by physical distance and connectivity (Martiny et al. 2006). This type of observation about inter- and intratumor genetic heterogeneity can potentially be associated with early and late stages of the disease, respectively. Such genetic data provide a way to infer the order of mutation occurrence and clone phylogenies (Miura et al. 2020) and the impact of specific treatment strategies on changing the tumor microenvironment and causing resistance (Sun 2016; Son et al. 2017; Witkowski et al. 2020).

Of course, molecular evolution is driven by random mutations and genetic drift. By random chance, genetic differences will accumulate across the genomes of cancer cells in every tumor of the patient’s ecosystem, causing genomic divergence and tumor heterogeneity. This leads to our third hypothesis that the observed tumor heterogeneity and divergence is primarily due to stochastic processes operating within tumors on existing and newly generated genetic variation. That is, the tumor area relationship, distance, and connectivity should have less influence on shaping tumor heterogeneity than random genetic drift and selection within tumors (fig. 2). In this hypothesis, tumor heterogeneity would be due to stochasticity in the fate of clones migrating and establishing metastases. That is, observed patterns of clone seedings and tumor sources between patients and across cancer types are idiosyncratic (Birkbak and McGranahan 2020).

Testing these above hypotheses about tumor insularity will ideally require spatial (multiple regions and tumors) and longitudinal (over the course of the disease) sequencing data, along with information about the size and distances between tumor sites. Tumor data manipulation and interpretation will also require much attention because tumor data often suffer from incomplete sampling of clones per tumor region and anatomical location (Kumar et al. 2020). This can potentially lead to incomplete and erroneous inferences of heterogeneity on tumor islands, producing false and inaccurate heterogeneity patterns.

Further limitations might also arise due to a low number of sampled genetic variants or errors in tumor sequencing data (Miura, Huuki, et al. 2018), because these can generate artifacts as well. However, we expect an abundance of higher quality and single-sequencing tumor data to be soon available, as we notice that more and more research consortia are collecting extensive tumor “multi-omics” data. Sampling tumor data to such extent and depth might be challenging now and be still in its infancy, but it will soon not be anymore, and they will undeniably be informative for investigating cancer evolution and tumor insularity.

We anticipate that exploring and validating the three hypotheses proposed here will enable us to leverage unique features of tumors to interrogate patterns of tumor heterogeneity among patients and across cancer types. Moreover, validation of the first hypothesis will not necessarily reject the second hypothesis. This is because variation on tumor heterogeneity patterns could arise due to the size and carrying capacity of the tumor and the distance and connectivity between tumors. However, rejection of both these hypotheses could lead to validation of the third hypothesis and the detrimental effect of stochastic processes on tumor heterogeneity. Ultimately, modeling and predicting tumor insularity will enable finding cohesion and establishing shared and unique features among tumors and their heterogeneity, accommodating the spatial and environmental dimensions of tumors that are being ignored or not so well understood.

## Using Methods of Organismal Biogeography to Map Metastatic Migrations

Several methods and frameworks in organismal biogeography explore the physical distance and species–area relationship while inferring origin and migration routes. These approaches leverage phylogenetic and evolutionary information and the location and sampling time information of the sampled sequences. Popular biogeographic methods include Bayesian Binary MCMC (BBM), BayArea, Dispersal Extinction Cladogenesis (DEC), and Dispersal-Vicariance Analysis (DIVA) (Ronquist and Huelsenbeck 2003; Ree and Smith 2008; Yu et al. 2010; Landis et al. 2013) (table 1).

**Table 1:** Matching Tumor Evolutionary Processes to Biogeographic Events and Existing Biogeographic Methods Would Provide More Insights into Cancer Cells’ Evolution and Migration

Tumor Evolutionary Processes	Biogeographic Events	Properties That Define Islands	Biogeographic Methods			
			BBM	BayArea	DEC	DIVA
Mutation (genetic divergence)	Duplication	n/a	Yes	No	Yes	Yes
Extinction	Extinction	n/a	Yes	Yes	Yes	Yes
Founder-event	Founder-event effect	n/a	No	Customize	Customize	Customize
Migration	Expansion	n/a	Yes	Yes	Yes	No
	Distant dispersal	n/a	Yes	Yes and distance-dependent effect on the dispersal probability	Yes	Yes
n/a	Vicariance	n/a	No	No	Yes	Yes
Sampling time or mutation rate	Time	n/a	Customize	Customize	Customize	No
n/a	n/a	Area size	Customize	No	No	No
n/a	n/a	Isolation (distance between areas)	Customize	No	No	No

Recent studies showcase the usefulness of applying biogeographic approaches to exploring cancer cells’ origin and evolutionary trajectories (Alves et al. 2019; Chroni et al. 2019, 2021). More specifically, Chroni et al. (2019) assessed the accuracy of a biogeographic approach (BBM) for inferring clone migration histories. Interestingly, they showed that it performed better than a method that has been specifically developed to infer clone migration patterns among tumors (MACHINA method) (El-Kebir et al. 2018). Alves et al. (2019) also explored applying a biogeographic method (BayArea) on clone migration inferences. More specifically, they found evidence of the effect of physical distance on the dispersal ability of tumor clones on a patient with colorectal cancer.

Another interesting aspect of the application of biogeographic methods and frameworks in cancer is that these enable conceptualizing and modeling processes that are critical to consider while inferring the origin and trajectory of migrations: duplication (genetic divergence within an area), extinction, founder events, and migration (dispersal, expansion, and vicariance) (table 1). These processes can be applied for describing the evolution and migration of cancer cells because cancer cells are also subject to dispersal, genetic divergence, extinction, and founder effect events (Chroni et al. 2021).

## Comparative Genetic Studies Show That Tumors Experience Insularity

We next discuss recent studies that reveal a spatial and temporal separation between tumors within a patient and how such evidence might indicate insularity in tumors. Recent data analyses of large cancer patient cohorts have reported extensive tumor genomic, histological, and phenotypic heterogeneity in different cancer types. Their review for their relevance to evidence of tumor insularity prompted us to categorize tumor ecosystems into islets, islands, and Archipelagos (box 1). Because cancer progresses over time and tumors are formed and cured in a patient, we anticipate that the tumor ecosystem may transform from one type into another. For example, cancer disease may begin in a patient as a tumor islet that becomes islands and forms an archipelago of tumors.

### Tumor Islets

Many cancer patients present with solitary primary tumors. We refer to these cases of tumor ecosystem as tumor islets because of the absence of recipient areas. Such cases have been reported in breast cancer (Caswell-Jin et al. 2019), Barrett’s esophagus cancer (Martinez et al. 2016; Yan et al. 2019), and liver cancer (Ding et al. 2019). These studies show intratumor heterogeneity due to spatially and temporally separated areas within primary tumors.

### Tumor Islands

Primary tumors and at least one surrounding or distant metastases are often identified in patients at the time of the first diagnosis. These patient cases will appear with slightly more complex tumor ecosystems with one or more recipient areas with limited connectivity between anatomical sites, which we call tumor islands. In these patient cases, we anticipate that tumor heterogeneity will be affected by the distance and degree of connectivity between anatomical sites. Indeed, studies show tumor heterogeneity to be the outcome of spatial and temporal separation between tumors in lung cancer (Jamal-Hanjani et al. 2017; Joshi et al. 2019), prostate cancer (Lindberg et al. 2015), and renal cancer (Turajlic, Xu, Litchfield, Rowan, Horswell, et al. 2018). Moreover, recent studies in pancreatic cancer discuss extensive morphological and histological heterogeneity attributed to the spatial and/or temporal separation of tumors with no indication of a connection between tumors (Chan-Seng-Yue et al. 2020; Hayashi et al. 2020).

Similarly, studies on nonsmall cell lung cancer (NSCLC) show tumors exhibiting spatial and temporal heterogeneity and extensive isolation (Diaz et al. 2012; Govindan et al. 2012; Swanton 2012; Jamal-Hanjani et al. 2014). In these studies, the observed temporal dynamics of tumor subclones were “tagged along” with branches of late mutagenic processes or specific mutations at later stages of the disease. On the other hand, Joshi et al. (2019) observed spatial heterogeneity of T cell antigen receptors (TCR) in NSCLC because TCR heterogeneity was correlated to their presence and mutational profiles of T cells in all and a subset of tumor regions (ubiquitous and regional, respectively). They also found that the observed heterogeneity in the T cells was correlated to the heterogeneity of clones, with this being also tumor regional specific (Joshi et al. 2019).

### Tumor Archipelagos

Cancer patients frequently manifest multiple tumors at proximate and distant anatomical sites. The degree of connectivity between these anatomical sites, for example, via lymph nodes, may facilitate the migration of cancer cells and contribute to tumor heterogeneity. In these instances, tumors with both source and recipient areas (primary and at least two metastatic tumors) with an extensive degree of connectivity are present in the form of back-and-forth clone migrations. So, we expect a higher degree of complexity in tumor ecosystems that would lead to the formation of tumor Archipelagos.

Indeed, there has been an ongoing discussion about the role of lymph nodes in formulating new metastases as in the metastatic progression in some cancer types. For example, a study on lung cancer showed evidence of correlation of lymph nodes to tumor relapse in specific histological subtypes and temporal isolation between primary and recurrent tumors during the progression of the disease (Abbosh et al. 2017). Two recent studies in clear-cell renal cell carcinoma also reported spatial and temporal isolation between metastases with evidence of the implication of tumor thrombus, lymph nodes, and adrenal glands (Turajlic, Xu, Litchfield, Rowan, Chambers, et al. 2018; Turajlic, Xu, Litchfield, Rowan, Horswell, et al. 2018). Other studies on pancreatic cancer (Hayashi et al. 2020), prostate cancer (Hieronymus et al. 2014; Gundem et al. 2015; Hong et al. 2015; Robinson et al. 2015), and colorectal cancer (Reiter et al. 2020) also show appealing evidence of the presence of tumor Archipelagos.

## Concluding Remarks and Future Perspectives

There is a growing body of evidence supporting the presence of high tumor heterogeneity within and among patients in all cancer types (Mroz and Rocco 2017; Williams et al. 2019; Birkbak and McGranahan 2020). At the same time, investigations focus on identifying genes, driver mutations, mutational signatures, and pathways involved in the initiation and progression of the specific cancer type or propose approaches to estimate tumor heterogeneity. These studies are also revealing extensive similarities and differences across patients within the same cancer type. Here, we argue that one way of conceptualizing all these properties is through the theoretical framework of tumors as evolutionary systems experiencing insularity. This conceptualization produces hypotheses and predictions about the effect of tumor insularity on tumor heterogeneity arising during metastatic disease progression. These hypotheses will enable researchers to investigate the relationship of the size and carrying capacities between tumors, the degree of physical distance between anatomical sites, and the impact of stochastic processes on tumor heterogeneity and metastatic potential. These will be possible to investigate because researchers will continue to examine tumor heterogeneity by exploring the interplay between “omics” data in a multidimensional scale of spatial (multiple regions and sites) and longitudinal (over the course of the disease) tumor data. In the future, engaging tumor insularity into mathematical models and frameworks that leverage the genetic and epigenetic aberrations will be key to investigating the relationship between tumor heterogeneity and microenvironment, exploring the metastatic potential, and even informing treatment design.
